# Supplementary figures and images for: Leishmania differentiation requires ubiquitin conjugation mediated by a UBC2-UEV1 E2 complex
Source: PLoS Pathog. 2020 Oct 27;16(10):e1008784. doi: 10.1371/journal.ppat.1008784 (PMC7647121; doi:10.1371/journal.ppat.1008784)

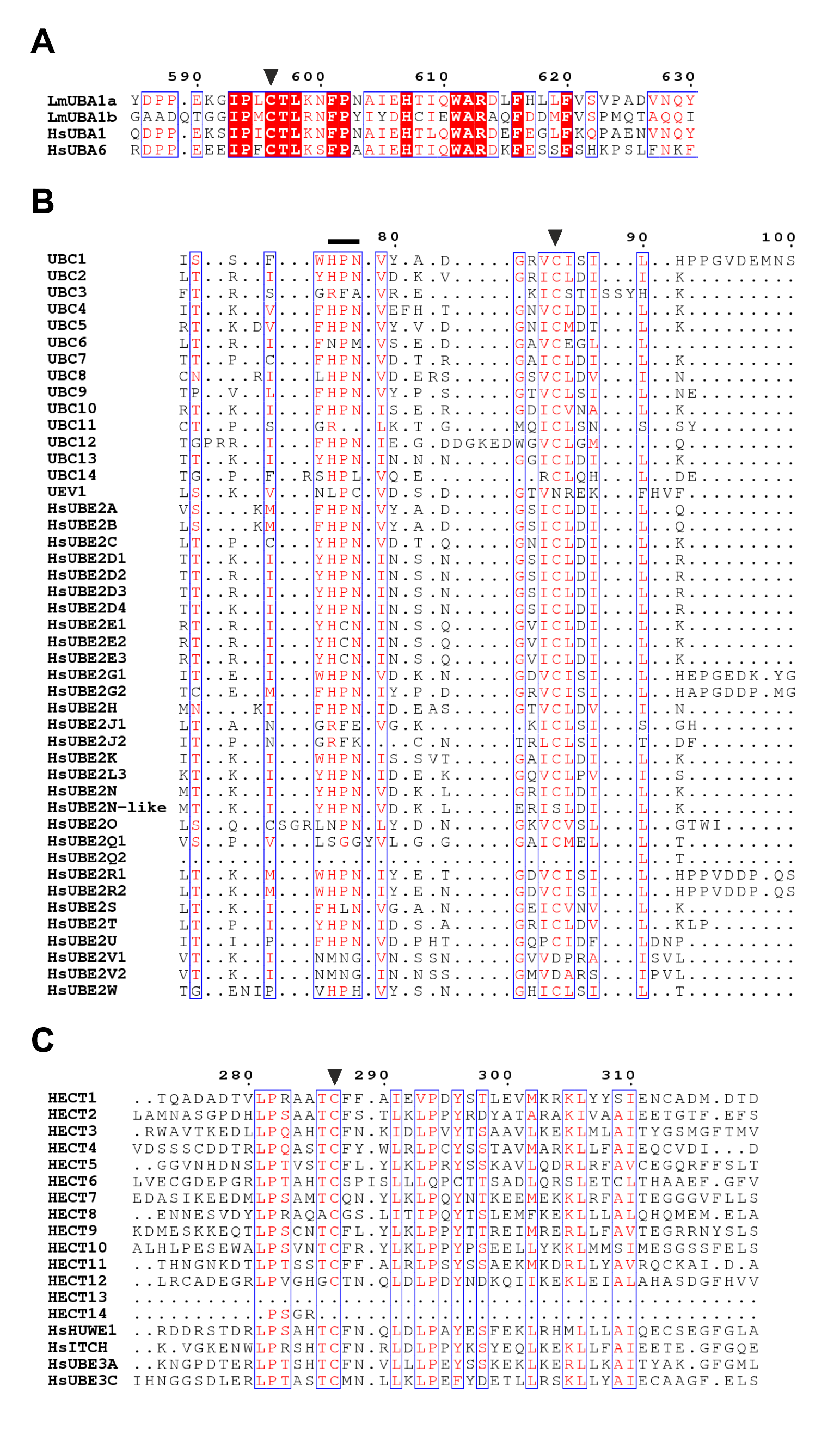

Supplement: S1 Fig — Sequences obtained from TriTryDB (L. mexicana) and NCBI or UniProt (H. sapiens) were aligned using T-Coffee. Residues in the local vicinity of the putative active site cysteine of A ubiquitin E1-activating enzymes, B E2-conjugating enzymes and C E3 ligase HECT domains are shown. Red boxes indicate amino acid identity, red characters show similarity within the highlighted group and blue frames highlight similarity across groups. Black bar indicates the position of the conserved HPN motif in E2 genes and the black triangles highlight the conserved catalytic cysteine residues in all classes of protein. L. mexicana genes are indicated with the prefix Lm and H. sapiens genes with the prefix Hs. Where no prefix is given, sequences belong to L. mexicana. (TIF) [file ppat.1008784.s005.tif]

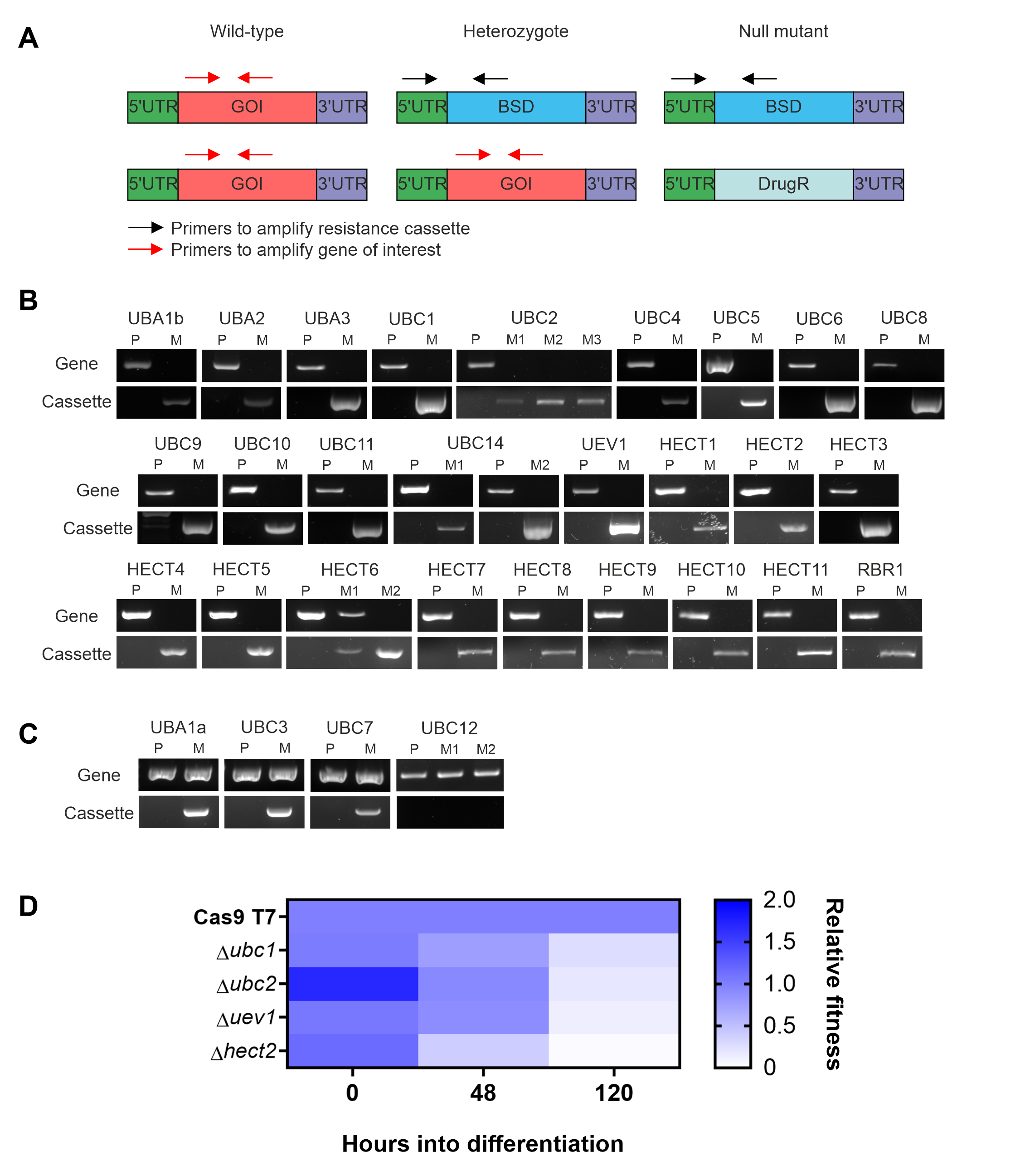

Supplement: S2 Fig — A Schematic of PCRs performed to identify null mutants from heterozygotes or mutants with additional gene copies. Primers were designed to amplify regions within the gene of interest (GOI, red primers) and between the 5’-UTR of the gene and the blasticidin repair cassette of the edited DNA (black primers). B PCRs performed on genomic DNA from parental (P) and mutant (M) cells are shown for genes that were deleted successfully. Due to the large size of HECT3 (18.6 kbp), only the first 8 kbp of the gene was targeted for editing. The HECT3 gene PCR was designed to amplify within the targeted region. C PCRs performed on genomic DNA from parental (P) and mutant (M) cells are shown for genes that could not be deleted (no mutant clones were obtained for UBC13). D Relative viability of Δubc1, Δubc2, Δuev1 and Δhect2 compared to the parental Cas9 T7 line during promastigote to amastigote differentiation. Time elapsed since the initiation of differentiation is marked on the x-axis. Data are an average of two independent experiments, each with 6 biological replicates. (TIF) [file ppat.1008784.s006.tif]

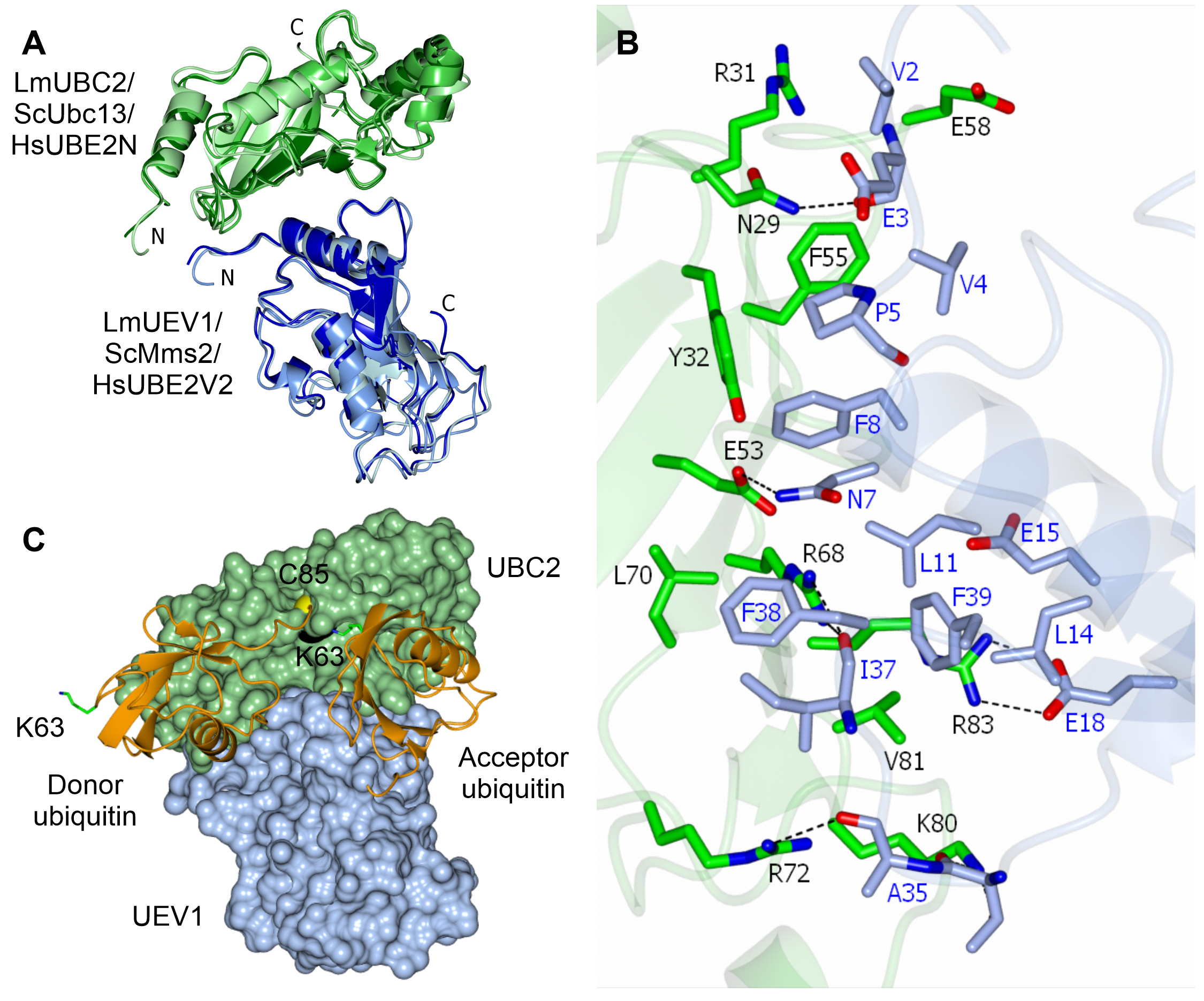

Supplement: S3 Fig — A Superposition of chains A and B for UBC2-UEV1 (dark green and dark blue, PDB ID: 6ZM3), HsUBE2N-UBE2V2 (darker green and darker blue, PDB ID: 1J7D) and ScUbc13-Mms2 (light green and light blue, PDB ID: 1JAT). Locations of the N- and C-termini are shown. B Zoom-in of the interface between UBC2 and UEV1 showing residues thought to contribute most significantly to complex formation according to analysis in the program PISA [97]. Residues are labelled in black for UBC2 and blue for UEV1. Residues are coloured by atom (red, oxygen; blue, nitrogen). Hydrogen bonds are denoted by dashed lines. C Superposition of UBC2-UEV1 onto the structure of the UBE2N-UBE2V2-Ub complex (PDB ID: 2GMI) [51] showing UBC2 (green) and UEV1 (blue) as space fill models and the positions of acceptor and donor ubiquitins (orange ribbons) obtained from the UBE2N-UBE2V2-Ub structure. C85 is represented by black (carbon) and yellow (sulfur) spheres. K63 of ubiquitin is highlighted as cylinders coloured by atom (blue, nitrogen). (TIF) [file ppat.1008784.s007.tif]

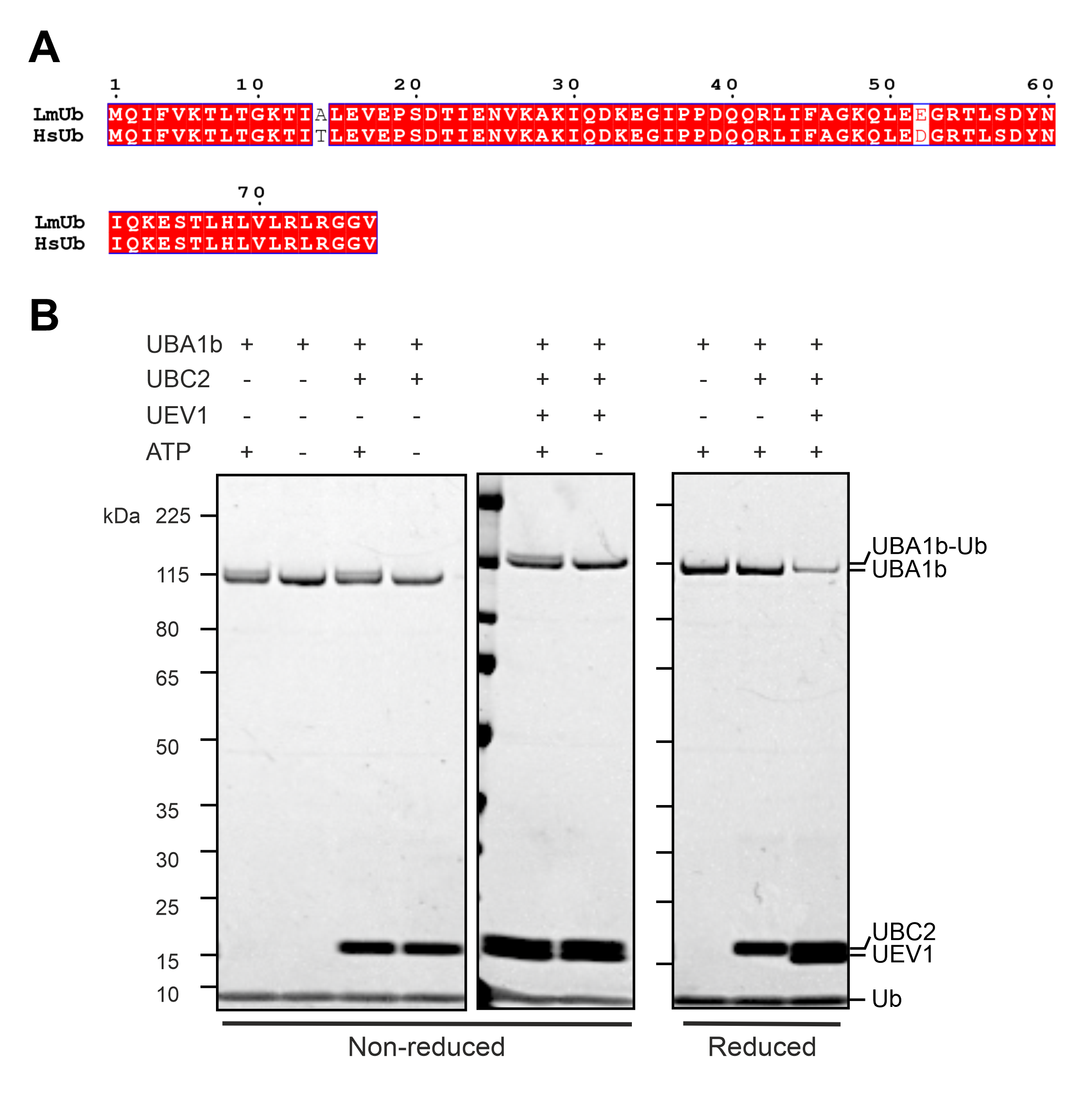

Supplement: S4 Fig — A Alignment of L. mexicana and H. sapiens ubiquitin protein sequences. Red boxes indicate amino acid identity, red characters show similarity within the highlighted group and blue frames highlight similarity across groups. B UBA1b and ubiquitin were incubated with UBC2, UEV1 and ATP as indicated in ubiquitination assay buffer for 30 min at 30°C. Samples were treated with either reducing or non-reducing sample buffer and visualised by SDS-PAGE with InstantBlue stain. (TIF) [file ppat.1008784.s008.tif]
